# Oleoylethanolamide as a New Therapeutic Strategy to Alleviate Doxorubicin-Induced Cardiotoxicity

**DOI:** 10.3389/fphar.2022.863322

**Published:** 2022-04-20

**Authors:** Yeyu Qin, Jing Xie, Ruihe Zheng, Yuhang Li, Haixia Wang

**Affiliations:** ^1^ Department of Pharmacy, Hainan General Hospital (Hainan Affiliated Hospital of Hainan Medical University), Haikou, China; ^2^ Medical College, Xiamen University, Xiamen, China; ^3^ Xiamen Institute of Rare-Earth Materials, Haixi Institutes, Chinese Academy of Sciences, Xiamen, China; ^4^ Department of Medical Oncology, Hainan General Hospital (Hainan Affiliated Hospital of Hainan Medical University), Haikou, China

**Keywords:** oleoylethanolamide (OEA), doxorubicin (DOX)-induced cardiotoxicity (DIC), peroxisome proliferator activated-receptor α (PPARα), transient receptor potential cation channel vanilloid-1 (TRPV1), PI3K/ akt signaling oleoylethanolamide (OEA), PI3K/ akt signaling

## Abstract

Doxorubicin (DOX) is one of the most common chemotherapeutic anti-cancer drugs. However, its clinical use is restricted by serious cardiotoxicity. Oleoylethanolamide (OEA), a structural congener of endocannabinoid anandamide, is the endogenous agonist of peroxisome proliferator activated-receptor α (PPARα) and transient receptor potential cation channel vanilloid-1 (TRPV1), and involved in many physiological processes. The present study aimed to determine whether OEA treatment protects against DOX-induced cytotoxicity (DIC) and gain insights into the underlying mechanism that mediate these effects. Our data revealed that Oleoylethanolamide treatment improved the myocardial structure in DOX-challenged mice by attenuating cardiac oxidative stress and cell apoptosis. OEA also alleviated DOX-induced oxidative stress and apoptosis dysregulation in HL-1 cardiomyocyte. These effects were mediated by activation of TRPV1 and upregulation of PI3K/ Akt signaling pathway. Inhibition of TRPV1 and PI3K reversed the protective effects of OEA. Taken together, our data suggested that OEA protects against DIC through a TRPV1- mediated PI3K/ Akt pathway.

## Introduction

Doxorubicin (DOX), a DNA topoisomerase II inhibitor, is commonly used for the treatment of a wide variety of cancers, including carcinomas, sarcomas and hematological cancers (Renu et al., 2018; Al-Malky et al., 2020). However, the clinical use of DOX is limited by cumulative cardiotoxicity, which can cause irreversible myocardial injury, manifested as a series of pathological changes including cardiomyopathy, arrhythmia, cardiac insufficiency and even heart failure in some cases (Renu et al., 2018). Although the specific regulatory mechanism of DOX-induced cardiotoxicity (DIC) remains unclear, it is thought to involve oxidative stress, fibrosis, autophagy and apoptosis dysregulation (Renu et al., 2018). To date, there are a limited number of drugs (e.g., dexrazoxane) that can be used for alleviating DIC (Seifert et al., 1994; Al-Malky et al., 2020). New therapeutic approaches that could prevent and treat DIC are still highly desired for patients.

Oleoylethanolamide (OEA) is a structural congener of endocannabinoid anandamide (Bowen et al., 2017). It is generated on-demand by small intestinal enterocytes and its production is stimulated by food intake (Fu et al., 2003). OEA can interact with different receptors in animals, including peroxisome proliferator activated-receptor α (PPARα) and transient receptor potential cation channel vanilloid-1 (TRPV1) (Ahern, 2003; Fu et al., 2003). In fact, as a drug, OEA modulates many physiological processes, including food intake, inflammation, neuro-protection, lipid metabolism and atherosclerotic plaques development *via* the activation of the PPARα signaling (Fu et al., 2003; Zhou et al., 2012; Yang et al., 2016; Bowen et al., 2017; Wu et al., 2019). Besides PPARα, OEA was suggested to act also as a medium-potency agonist of TRPV1 (Ahern, 2003). However, the role of TRPV1 in OEA’s actions remains less-well known.

OEA play beneficial roles in inflammation, apoptosis and oxidative stress related diseases (Pandey et al., 2009), opening up the possibility that OEA would have a therapeutic effect on DIC. In the current study, we examined whether treatment of OEA could attenuate DIC. We found that OEA significantly alleviated DOX-induced cardiac oxidative stress, cell apoptosis and tissues damage *in vivo* and *in vitro*. These therapeutic effects could be blocked by TRPV1 antagonist capsazepine and PI3K inhibitor LY294002. Our results identified OEA as a promising therapy for DIC.

## Methods

### Drugs

Doxorubicin was purchased from Macklin Biochemical (Cat. #D807083). OEA (Cat. #Z130746), LY294002 (Cat. #L124970), fenofibrate (Cat. #F129682), capsazepine (Cat. #C126558) was purchased from Aladdin. GW6471 (Cat. # 11697) and nonivamide (Cat. # 25328) was purchased from Cayman chemical.

### Animals and Treatments

The animal experiments were approved by the Institutes of Health and approved by the Animal Care and Use Committee of Hainan Medical University. C57BL/6 male mice (20–25 g) were purchased from the Vital River Laboratory Animal Technology Co., Ltd. Mice were group-housed in ventilated cages with controlled temperature (25 ± 1°C), relative humidity (55 ± 10%) and a 12-h day/night cycle at 20–25°C. Standard mouse chow and tap water were provided ad libitum.

The DIC model was reproduced as previously reported (Tadokoro et al., 2020). *N* = 6 mice for control and each DIC group. Briefly, DOX (6 mg/kg) or its vehicle was intravenously administered to mice at 9:00 a.m. at days 1, 2, and 4. Mice were intraperitoneally treated with OEA (15 or 30 mg/kg) at 2:00 p.m. (Fu et al., 2003). once daily, PI3K inhibitor LY294002 (10 mg/kg) once daily, or TRPV1 antagonist capsazepine (5 mg/kg) at 6:00 p.m. once daily for 14 consecutive days. All mice were then sacrificed at day 15 and the heart was excised and collected.

### Histological Analysis

Hearts were excised, sectioned, fixed in 10% (w/v) formalin for 24 h, followed by embedding in paraffin. The specimen was embedded in paraffin, cut in 5 μm sections and stained with Masson’s trichrome, and were photographed using a digital microscope at the magnification of ×400 (Sun et al., 2020). The severity of fibrosis was determined by the previous reported method (Sun et al., 2020). Total three sections per heart were analyzed. The score was calculated from three different and nonoverlapping fields in each section, and the average value was taken for each heart. The trained operator and data analysis were blinded to the experimental groups.

### Immunofluorescence

Immunofluorescence staining was performed according to standard protocols (Sun et al., 2020; Li et al., 2021). The paraffin embedded heart sections and cell slides were incubated with the following primary antibodies at 4C overnight: goat anti-8-hydroxy-2′-deoxyguanosine (8-OHdG) (Sigma, Cat. # AB5830, 1:500 dilution) and rabbit anti-caspase-3 antibody (Abcam, Cat. # ab32351, 1:500 dilution). After washing, sections were incubated with donkey anti-goat IgG 647 (Abcam, Cat. # ab150135, 1:1,000 dilution) or goat anti-rabbit IgG 555 (Abcam, Cat. # ab150078, 1:1,000 dilution) at room temperature for 2 h, and counterstained with 1 μg/ml DAPI. All fluorescence images were obtained using the Nikon Eclipse Ti-U fluorescence microscope and analyzed from five randomly selected fields by ImageJ.

### TdT-Mediated dUTP Nick End-Labeling Staining

TUNEL staining was performed using the *in situ* Apoptosis Detection Kit (Takara Bio, Cat. # MK500) according to supplier’s protocol (Liang et al., 2020). Briefly, the paraffin-embedded heart tissue sections were deparaffinized in xylene, rehydrated in aqueous solutions with decreasing alcohol content, and digested with proteinase K at room temperature for 15 min. The slides were then incubated with the TUNEL reaction mixture at 37°C for 1 h in a humidified chamber, followed by incubation with 4′,6-diamidino-2-phenylindole (DAPI) for 10 min.

### Serum Biochemical and Immunochemical Analyses

Serum levels of cardiac troponin I (cTnI) (Nanjing Jiancheng Bioengineering Institute, Cat. #H149-2), heart-type fatty acid-binding protein (H-FABP) (Dldevelop, Cat. # DL-FABP3-Mu) and myoglobin (Dldevelop, Cat. # DL-MYO-Mu) were measured using corresponding enzyme-linked immunosorbent assay (ELISA) kits following the manufacturer’s instructions (Kalantary-Charvadeh et al., 2019). Serum creatine kinase isoform MB (CK-MB) level was measured by using a colorimetric assay kit (Sigma, Cat. # MAK116) following the manufacturer’s instructions (Kalantary-Charvadeh et al., 2019).

### Thiobarbituric Acid Reactive Substances

TBARS were determined using a previous reported method (Sahu et al., 2019). Heart samples were homogenized in ice-cold potassium phosphate buffer (3 ml, 50 mM, pH 6, containing 0.32 M sucrose) for 3 min, and the mixture were allowed to react with 0.5 ml of 3% sodium dodecyl sulfate, 2 ml of 0.1 N HC1, 0.3 ml of 10% phosphotungstic acid, and 1 ml of 0.7% 2-thiobarbituric acid at 90 °C for 30 min. The mixture was extracted with 5 ml L-butanol. After a brief centrifugation, the supernatants were collected and continuously scanned at a wavelength of 515 nm excitation and 555 nm emission. The values were defined as the amount of TBARS (malondialdehyde equivalents) per Gram of heart. Malondialdehyde standards were prepared from 1,1,3,3-tetramethoxypropane.

### Glutathione-S-Transferase

GST activity was determined using a fluorescent assay kit (Thermo Fisher, Cat. # EIAGSTF) following the manufacturer’s instructions (Sahu et al., 2019).

### Cell Culture and Treatment

HL-1 cardiomyocytes (Sigma, Cat. # SCC065) were cultured in supplemented Claycomb Medium (Sigma, Cat. # 51800C) in an incubator at 37°C with 5% CO_2_ atmosphere. Adult derived primary human cardiomyocytes (Celprogen, Cat # 36044-15) were cultured in complete growth media (Celprogen, Cat #M36044-15S) in an incubator at 37°C with 5% CO_2_ atmosphere. Cells were plated into 12-well plate and cultured until 80% confluence. Cells were then incubated with vehicle (0.1% DMSO), OEA (30 μM), capsazepine (10 μM), GW6471 (5 μM), nonivamide (10 μM), fenofibrate (100 μM), LY294002 (10 μM) for 30 min, or TRPV1 siRNA (5′-GUU​CGU​GAC​AAG​CAU​GUA​CTT-3′, 3′-TTC​AAG​CAC​UGU​UCG​UAC​AUG-5′, 30 nM) and HiPerfect transfection reagent (Qiagen, 301704) for 18 h before challenged by 2 μM DOX. After 24 h, cells were harvested for analysis (Liu et al., 2020).

For immunofluorescence staining, HL-1 cells were cultured on coverslips under the same conditions as described above. HL-1 cardiomyocytes were fixed in 4% paraformaldehyde and 0.4% Triton X-100 (Aladdin, Cat. #T109026) for 30 min at room temperature, respectively. The cells were then washed 3 times with PBS and blocked with goat serum (Boster, Cat. # AR0009) at 37 °C for 1 h before immunofluorescence staining (Zhou et al., 2019; Sun et al., 2020).

### Cell Proliferation Assay

Cell viability were measured using the cell counting kit-8 (CCK8) (Dojindo, Cat. # CK04) according to the manufacturer’s instructions (Liu et al., 2020). Cells were seeded into 96-well plates at a density of 1,000 per well and cultured under the same conditions as described above. Tetrazolium reagent (10 μl) provided from the kit was added into each well, and incubated at 37°C for 1 h. The plate was measured at a wavelength of 450 nm using a microplate reader. Percent cell proliferation was defined as the relative percentage (%) of treated cells relative to untreated control group.

### Western Blot

Western blotting was performed according to standard protocols (Tadokoro et al., 2020). The following antibodies were used: rabbit anti-PI3K antibody (Abcam, Cat. # ab86714, 1:1,000 dilution), rabbit anti-p-Akt antibody (Abcam, Cat. # ab222489, 1:1,000 dilution), rabbit anti-β-actin (Sigma, Cat. # A5441, 1:50,000 dilution). As secondary agents, horseradish peroxidase-coupled goat anti-rabbit antibody was used. Bands were visualized with an enhanced chemiluminescence detection kit (Thermo, WP20005). All results were quantified using ImageJ software, with β-actin as the internal standard.

### Statistical Analysis

The results are presented as the means ± SEM. One-way ANOVA was used to analyze the differences between experimental groups, followed by Tukey’s Multiple Comparison Test using GraphPad Prism 9.0 software. In all cases, *p* < 0.05 was considered to indicate a statistically significant difference.

## Results

### OEA Alleviates DOX-Induced Cardiotoxicity in Mice

**FIGURE 1:**
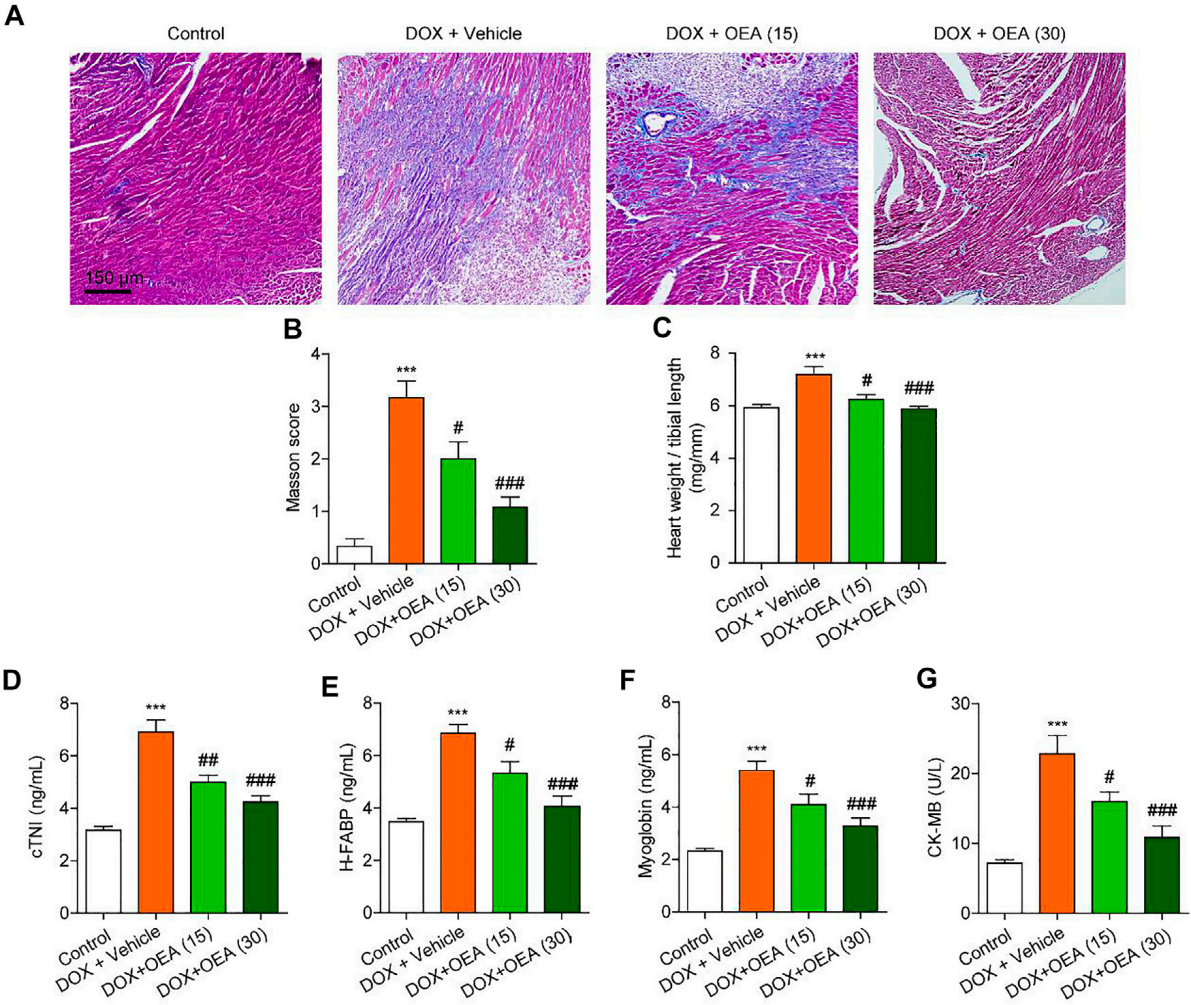
OEA alleviates DIC in mice. Mice with or without DOX (6 mg/kg, i. v.) challenge were treated with OEA (15 or 30 mg/kg, i. v.) for 14 days concurrently with DOX challenge. Heart tissues were isolated at day 15 for analysis. Representative histopathological sections of heart tissues by **(A)** Masson’s trichrome staining. **(B)** Myocardial fibrosis in the areas as described in **(A)** were graded. The effects of OEA or its vehicle on (C) heart weight/ tibial length ratio, serum levels of **(D)** cTNI **(E)** H-FABP **(F)** myoglobin and **(G)** CK-MB in mice. Data are expressed as mean ± SEM, *n* = 6. ***, *p* < 0.001 vs. control. #, *p* < 0.05; ##, *p* < 0.01; ###, *p* < 0.001 vs. DOX + vehicle.

We first assessed the potential protective effects of OEA on DIC. As shown in Figure 1A, Masson staining revealed that DOX challenge increased the degree of myocardial fibrosis, while OEA reduced the formation of fibrosis foci in the mice heart (Figures 1A–C). Furthermore, cTnI, H-FABP, myoglobin and CK-MB are sensitive markers of cardiomyocyte damage in the serum. DOX treatment elicited a drastic increase in the levels of cTnI, H-FABP, myoglobin and CK-MB in the serum of DIC mice, while OEA dose-dependently reduced the increment of these markers (Figures 1D–G). In addition, treatment of normal mice with OEA alone did not affect the histological features of heart and levels of cTnI, H-FABP, myoglobin and CK-MB (Supplementary Figure S1). Taken together, these data illustrate that OEA exhibits a protective role in DIC.

### OEA Inhibits DOX-Induced Cardiac Oxidative Stress and Apoptosis in Mice

**FIGURE 2:**
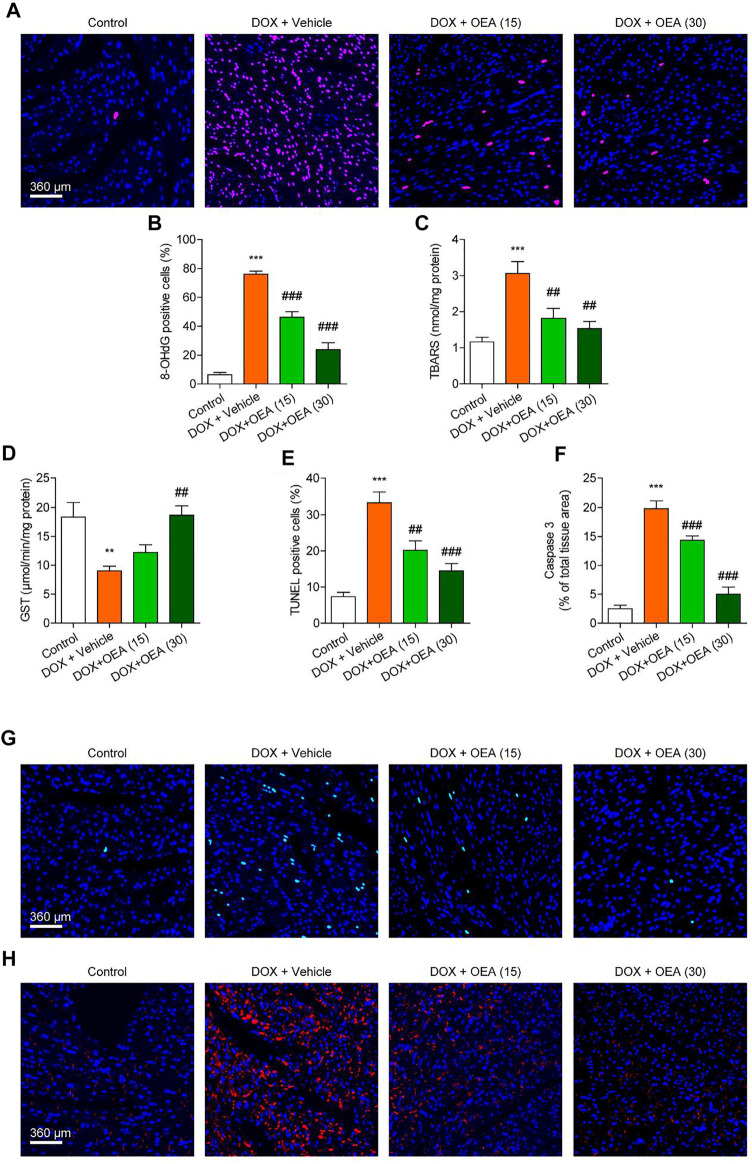
OEA inhibited DOX-induced myocardial oxidative stress and apoptosis in mice. Mice with or without DOX (6 mg/kg, i. v.) challenge were treated with OEA (15 or 30 mg/kg, i. v.) for 14 days concurrently with DOX challenge. Heart tissues were isolated at day 15 for analysis. **(A)** Immunofluorescence staining of 8-OHdG in myocardial tissues. **(B)** 8-OHdG positive cells shown in **(A)** were counted. The effects of OEA or its vehicle on **(C)** TBARS and **(D)** GST in myocardial tissues. **(E)** TUNEL positive cells shown in **(G)** were counted. **(F)** Fluorescence quantification for caspase three in **(H)** were calculated. **(G)** Detection of apoptotic cells in mice cardiac tissues by TUNEL staining. **(H)** Immunofluorescence staining of caspase three in myocardial tissues. Data are expressed as mean ± SEM, *n* = 6. ***, *p* < 0.001 vs. control. #, *p* < 0.05; ##, *p* < 0.01; ###, *p* < 0.001 vs. DOX + vehicle.

Dox chemotherapy has been reported to induce cardiotoxicity *via* lipids peroxidation and oxidative DNA damage in cardiomyocytes (Minotti et al., 1996). Cardiac TBARS and 8-OHdG are the markers of lipid peroxidation and oxidative DNA damage, respectively (Valavanidis et al., 2009; Aguilar Diaz De Leon and Borges, 2020). Dox caused significant up-regulation in expressions of 8-OHdG, a marker of oxidative DNA damage, and TBARS, a marker of lipid peroxidation, which were dose-dependently reduced by OEA treatment (Figures 2A–C). DOX challenge caused oxidative stress to the myocardial tissues *via* depletion of endogenous anti-oxidant enzyme GST, while OEA significantly restored activities of these anti-oxidant enzymes (Figure 2D). Cardiomyocyte apoptosis plays an important role in the pathophysiology of DIC. There were more TUNEL-positive cells in the heart of DOX-treated mice than in that of normal mice (Figures 2E,G). Simultaneous up-regulation of cleaved caspase three confirmed the cells apoptosis in the cardiac tissue of DOX-treated mice (Figures 2F,H). On the other hand, OEA treatment significantly attenuated DOX-induced cell apoptosis (Figures 2F–H). Overall, these observations suggested that OEA inhibited cardiac oxidative stress and apoptosis in DIC mice.

### OEA Inhibits DIC in HL-1 Cardiomyocytes Through a TRPV1-dependent Pathway

**FIGURE 3:**
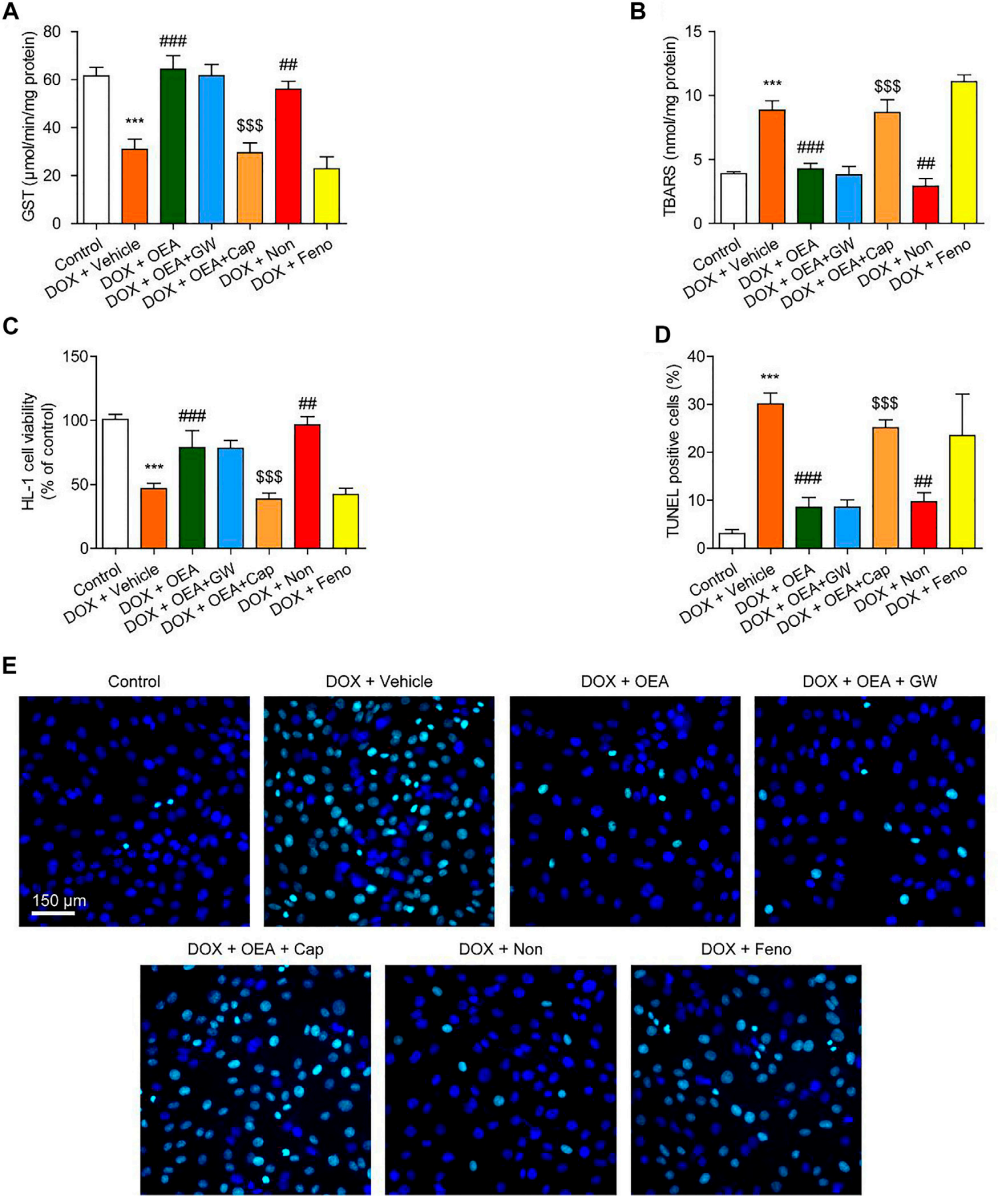
OEA inhibited DOX-induced oxidative stress and apoptosis in cardiomyocytes through TRPV1 pathway. HL-1 cells were treated with 0.1% DMSO, OEA (30 μM), GW6471 (5 μM), capsazepine (10 μM), nonivamide (10 μM), fenofibrate (100 μM) for 30 min. Cells were then treated with DOX (2 μM) for 24 h. Levels of **(A)** GST **(B)** TBARS and **(C)** cell viability was assessed in HL-1 cardiomyocytes. **(D)** TUNEL positive cells shown in **(E)** were counted. **(E)** TUNEL staining of HL-1 cardiomyocytes. Data are expressed as mean ± SEM, *n* = 4. ***, *p* < 0.001 vs. control. ##, *p* < 0.01; ###, *p* < 0.001 vs. DOX + vehicle. $$$, *p* < 0.001 vs. DOX + OEA.

We further investigated the mechanism whereby OEA prevents DIC in HL-1 cardiomyocytes. OEA is known as an agonist of PPARα and TRPV1 (Ahern, 2003; Fu et al., 2003), thus we investigated whether OEA prevents oxidative stress and cell apoptosis through PPARα and/ or TRPV1 pathways. Consistent with *in vivo* results, OEA treatment reduced cardiac oxidative stress in DOX-challenged HL-1 cells (Figures 3A,B). The protective effects of OEA were completely prevented by TRPV1 antagonist capsazepine, but not by PPARα antagonist GW6471 (Figures 3A,B). In addition, TRPV1 agonist nonivamide but not PPARα agonist fenofibrate improved cellular oxidative defense in HL-1 cells treated with DOX (Figures 3A,B). We also measured cells viability and apoptosis at 24 h after DOX challenge. Treatment of OEA increased cells viability and reduced cells apoptosis (Figures 3C–E). The beneficial effects of OEA were impaired after pre-treatment with TRPV1 antagonist capsazepine but not PPARα antagonist GW6471 (Figures 3C–E). To confirm the role of TRPV1 in OEA-mediated therapeutic effects, we also tested the actions of OEA in TRPV1- deficiency cells. We found that the effects of PEA on GST, TBARS and TUNEL were prevented by TRPV1 deletion in HL-1 cells transfected with TRPV1 siRNA (Supplementary Figures S2A–C). These results together with the data obtained from inhibitors studies indicated that OEA inhibited DIC through TRPV1 pathway activation.

### OEA Promotes TRPV1-Mediated PI3K/ Akt Signaling in HL-1 Cardiomyocytes

**FIGURE 4:**
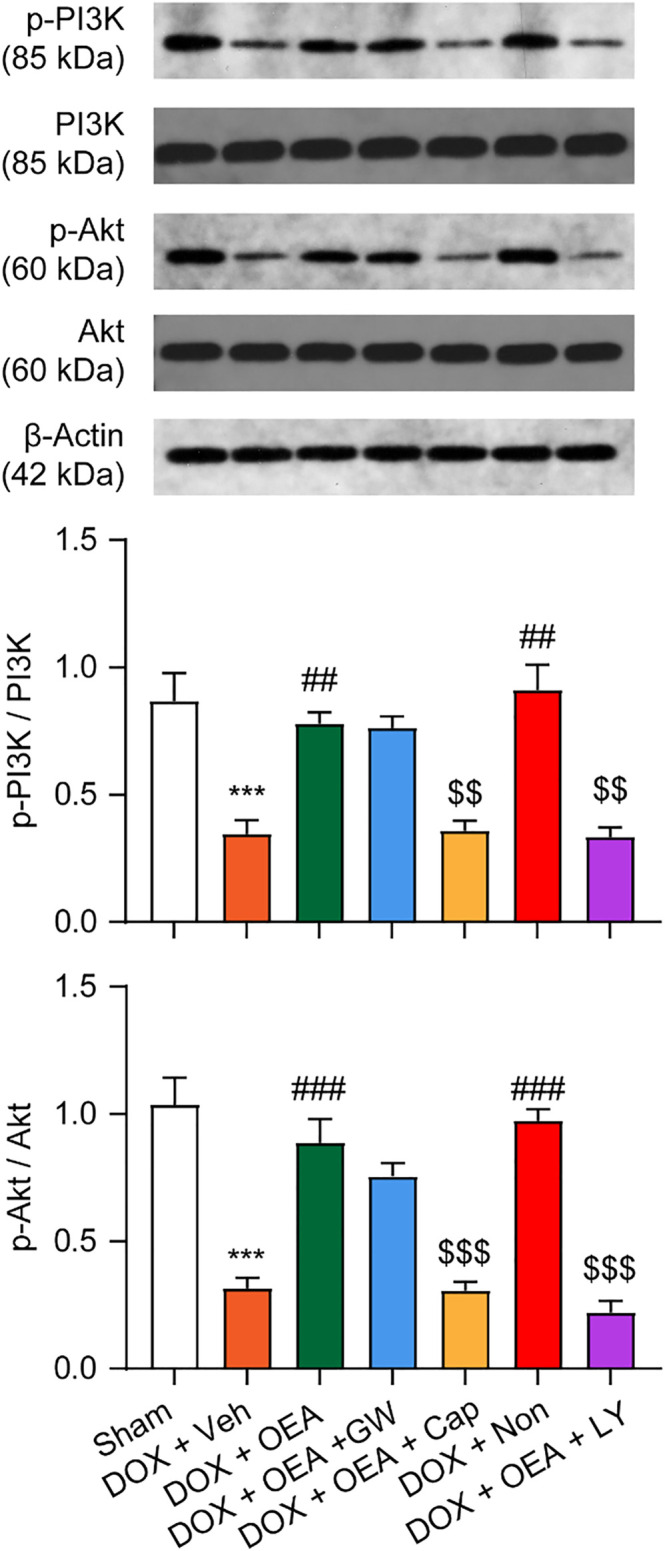
OEA promotes TRPV1-mediated PI3K/ Akt signaling in cardiomyocytes. HL-1 cells were treated with 0.1% DMSO, OEA (30 μM), GW6471 (5 μM), capsazepine (10 μM) and nonivamide (10 μM) for 30 min. Cells were then treated with DOX (2 μM) for 24 h. PI3K, Akt, p-PI3K and p-Akt was determined using western blot analysis.

TRPV1 is known to activate PI3K/Akt signaling and promote cells survival (Zhai et al., 2020). To verify that the PI3K/Akt signaling pathway activation is involved in the protective effects of OEA in DOX-treated cardiomyocytes, we examined PI3K and p-Akt expression on HL-1 cells that were cultured for 24 h in the absence or presence of OEA and/or the PI3K inhibitor, LY294002. As illustrated in Figure 4, DOX reduced PI3K expression in HL-1 cells, while OEA and nonivamide restored the PI3K expressions to normal levels. This effect of OEA was abolished by capsazepine. DOX also caused a reduction in cellular phosphorylated Akt (p-Akt) content, while treatment with OEA and nonivamide blocked the DOX-induced reduction of p-Akt levels. Inhibition of PI3K activity with LY294002 or antagonism of TRPV1 with capsazepine respectively reversed the effects of OEA. These data suggested that OEA promotes TRPV1-mediated PI3K/ Akt signaling in cardiomyocytes.

**FIGURE 5:**
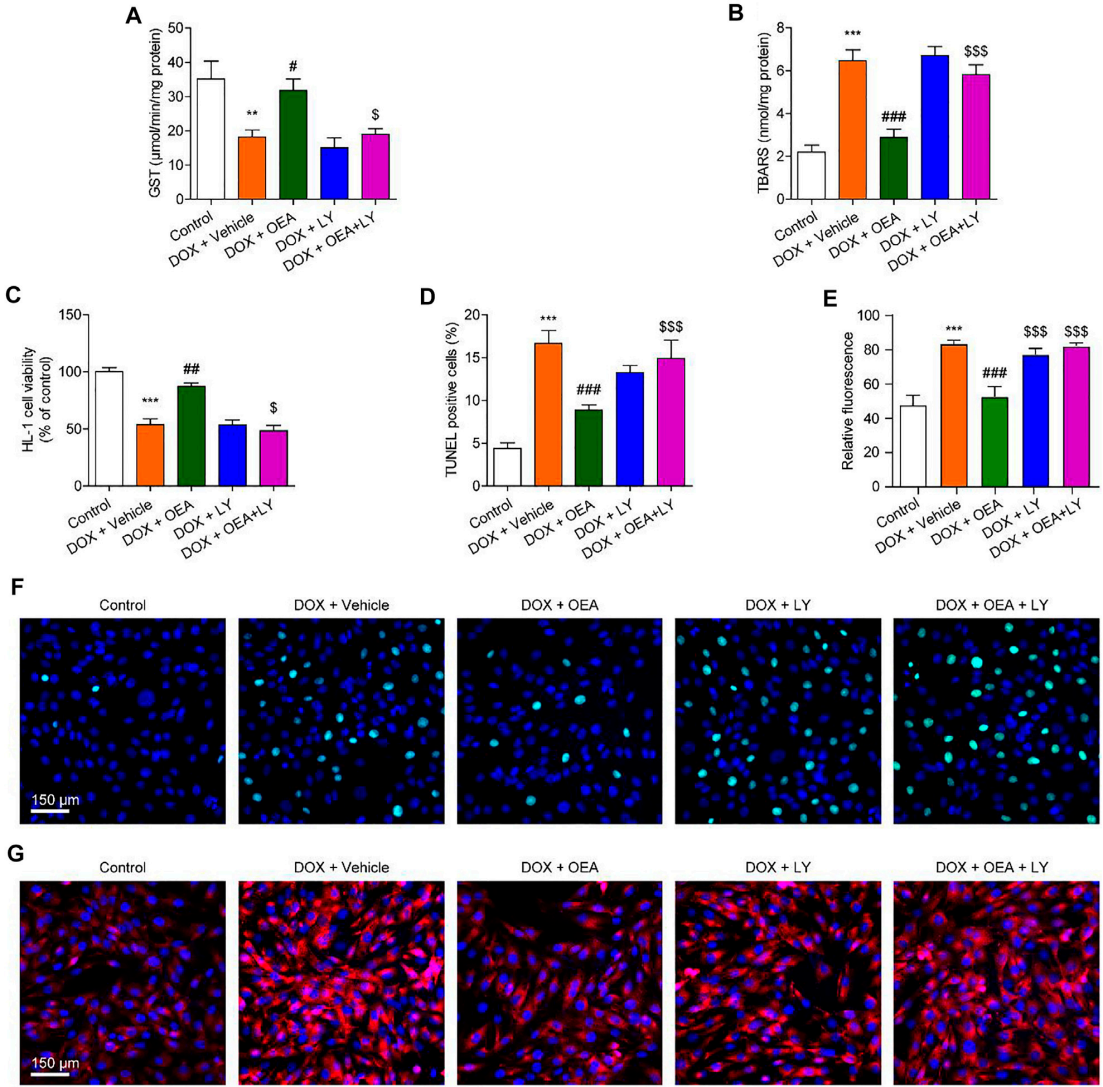
Inhibition of the PI3K-Akt signaling blocks the protective effects of OEA in cardiomyocytes. HL-1 cells were treated with 0.1% DMSO, OEA (30 μM) and LY294002 (10 μM) for 30 min. Cells were then treated with DOX (2 μM) for 24 h. Levels of **(A)** GST **(B)** TBARS and **(C)** cell viability was assessed in HL-1 cardiomyocytes. **(D)** TUNEL positive cells shown in **(F)** were counted. **(E)** Fluorescence quantification for caspase three in **(G)** were calculated. **(F)** TUNEL staining of HL-1 cardiomyocytes. **(G)** Immunofluorescence staining of caspase three in HL-1 cardiomyocytes. Data are expressed as mean ± SEM, *n* = 4. ***, *p* < 0.001 vs. control. #, *p* < 0.05; ##, *p* < 0.01; ###, *p* < 0.001 vs. DOX + vehicle. $, *p* < 0.05; $$, *p* < 0.01; $$$, *p* < 0.001 vs. DOX + OEA.

Next, we examined whether PI3K/ Akt signaling plays a role in the protective effects of OEA in HL-1 cardiomyocytes. As illustrated in Figures 5A,B, the antioxidant actions of OEA were reversed by PI3K inhibitor LY294002. Moreover, OEA failed to increase cells viability and reduce cells apoptosis in LY294002-treated HL-1 cardiomyocytes (Figures 5C–G). Additionally, we also confirmed these results in adult derived primary human cardiomyocytes. As shown in Supplementary Figure S3, OEA effectively reduced cardiac oxidative stress and cells apoptosis in DOX-challenged cardiomyocytes, while capsazepine and LY294002 prevented these effects. These *in vitro* data confirm the essential role of TRPV1-mediated PI3K/ Akt signaling in the protective effects of OEA.

### OEA Attenuates DIC in Mice Through a TRPV1-Mediated PI3K/ Akt Pathway

**FIGURE 6:**
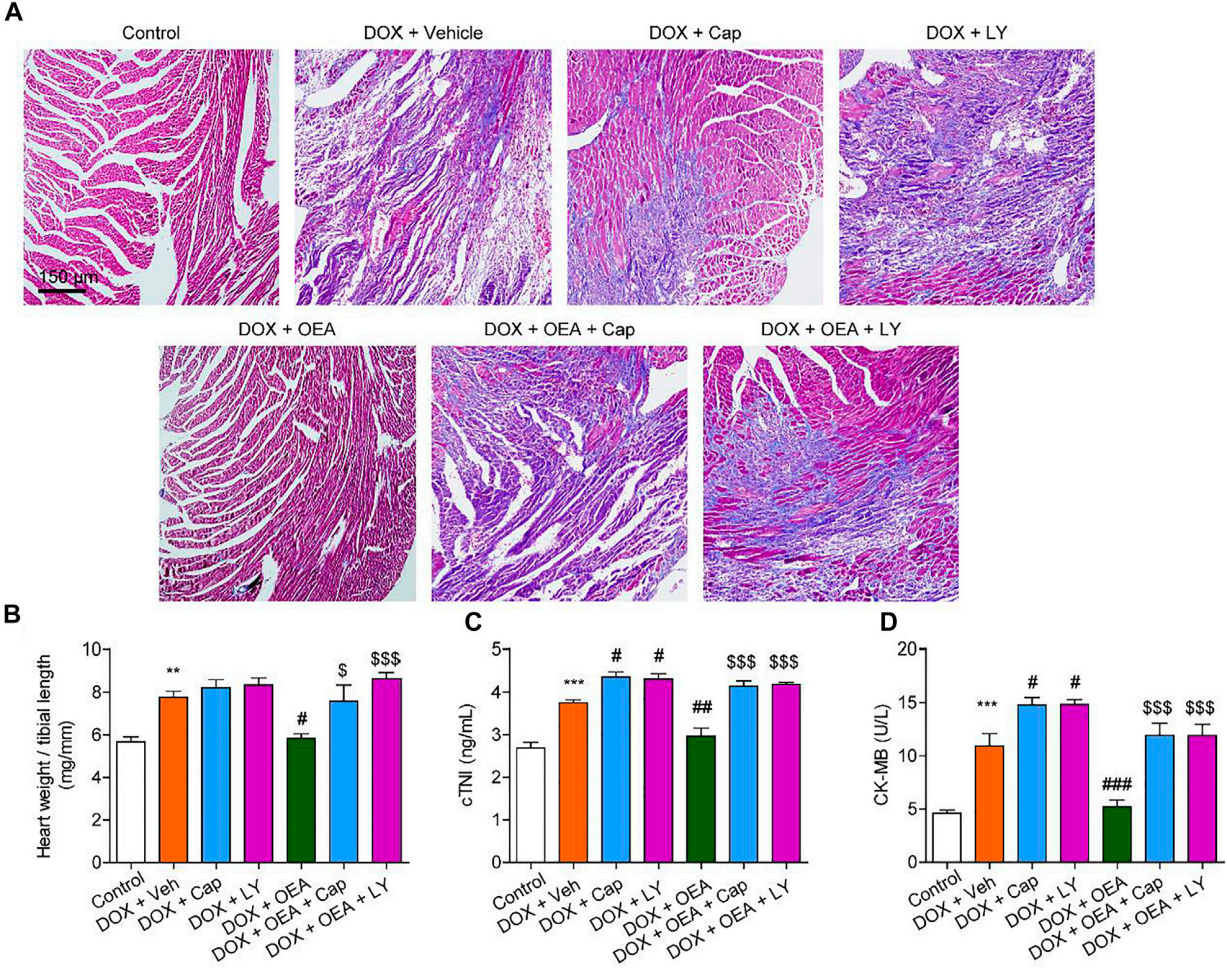
OEA attenuates DIC in mice through a PI3K-dependent pathway. Mice with or without DOX (6 mg/kg, i. v.) challenge were treated with OEA (30 mg/kg, i. v.), capsazepine (5 mg/kg, i. v.) and LY294002 (10 mg/kg, i. v.) for 14 days concurrently with DOX challenge. Heart tissues were isolated at day 15 for analysis. **(A)** Representative histopathological sections of heart tissues by Masson staining. The effects of OEA and LY294002 on **(B)** heart weight/ tibial length ratio, serum levels of **(C)** cTNI and **(D)** CK-MB in mice. Data are expressed as mean ± SEM, *n* = 6. ***, *p* < 0.001 vs. control. #, *p* < 0.05 vs. DOX + vehicle. $$$, *p* < 0.001 vs. DOX + OEA.

**FIGURE 7:**
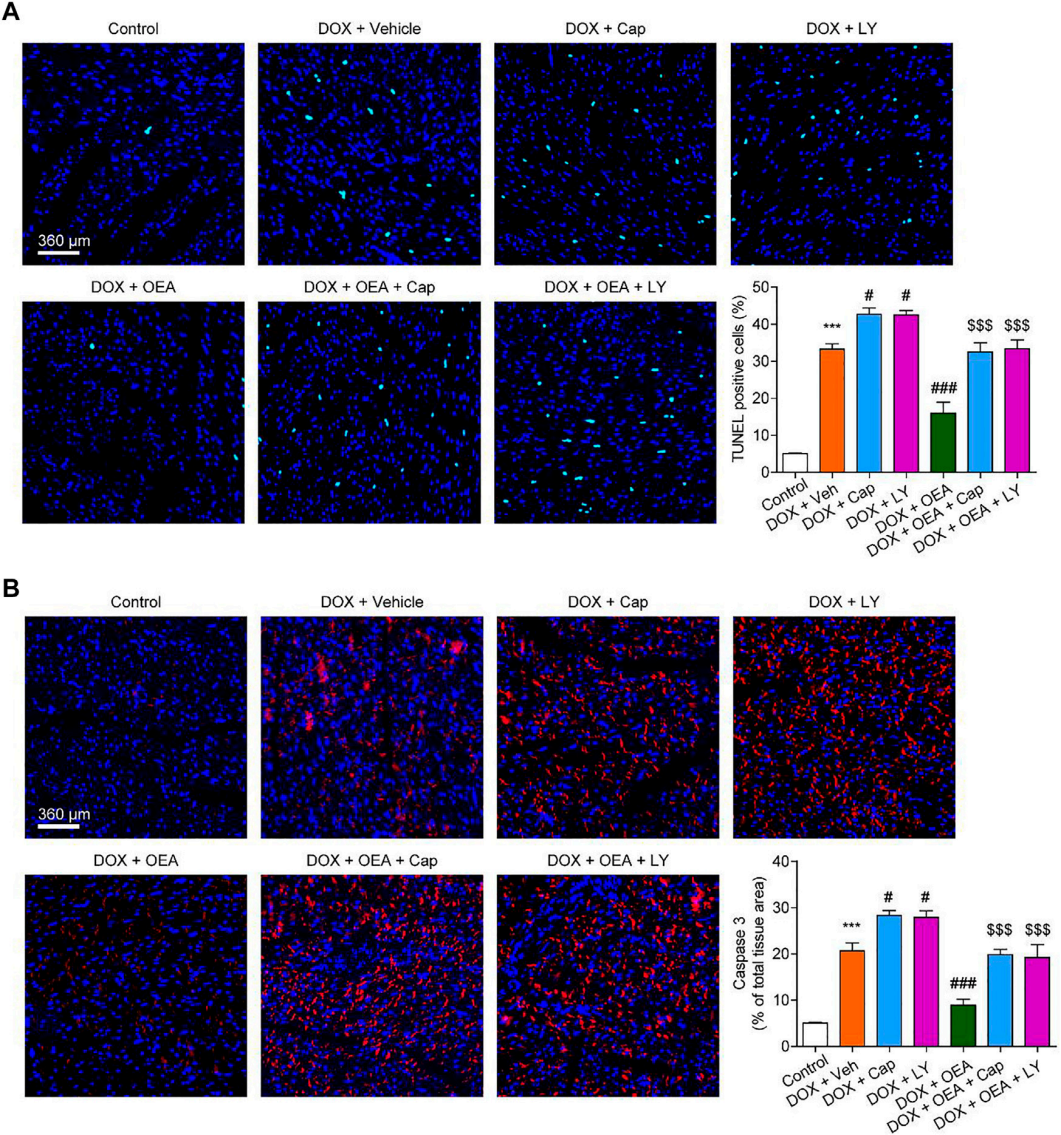
OEA attenuates DOX-mediated myocardial oxidative stress in mice through a PI3K-dependent pathway. Mice with or without DOX (6 mg/kg, i. v.) challenge were treated with OEA (30 mg/kg, i. v.), capsazepine (5 mg/kg, i. v.) and LY294002 (10 mg/kg, **(I)** v.) for 14 days concurrently with DOX challenge. Heart tissues were isolated at day 15 for analysis. **(A)** Detection of apoptotic cells in mice cardiac tissues by TUNEL staining. TUNEL positive cells were counted. **(B)** Immunofluorescence staining of caspase three in myocardial tissues. Expression of caspase three were calculated. Data are expressed as mean ± SEM, *n* = 6. ***, *p* < 0.001 vs. control. #, *p* < 0.05 vs. DOX + vehicle. $$$, *p* < 0.001 vs. DOX + OEA.

To further confirmed the role of TRPV1-mediated PI3K/ Akt signaling in OEA-mediated cardiac protection, we determined the effect of LY294002 and capsazepine in DOX-treated mice. As shown in Figures 6, 7, treatment with LY294002 or capsazepine alone augmented DIC in mice. Furthermore, inhibition of PI3K activity with LY294002 or antagonism of TRPV1 with capsazepine, reversed the protective effect of OEA against DIC in cardiomyocytes, as demonstrated by myocardial fibrosis (Figures 6A,B), cardiac tissue damage (Figures 6C,D) and increased number of apoptotic cells (Figure 7). In summary, these results revealed that OEA attenuates DIC in mice through a TRPV1-mediated PI3K/ Akt pathway.

## Discussion

DOX is one of the most widely used chemotherapeutic anticancer drug (Al-Malky et al., 2020). However, prolonged clinical use of this compound has been associated to multiple serious effects, especially cardiovascular toxicity (Al-Malky et al., 2020). Mechanisms by which Dox promotes cardiac injury remains controversial. Enhanced oxidative stress and apoptosis dysregulation have been proposed to be involved in Dox-mediated cardiomyopathy (Renu et al., 2018). In this study, we observed that OEA alleviated DOX-induced cardiac dysfunction and cell apoptosis *in vitro* and *in vivo*. Furthermore, the protective effect of OEA was dependent on TRPV1-mediated PI3K/ Akt pathway, as inhibition of TRPV1 and PI3K abolished such effect.

Oxidative stress responses, including myocardial lipids peroxidation, DNA oxidative damage and generation of excessive ROS, have been reported to contribute to DIC (Minotti et al., 1996). Oxidative stress can activate serval apoptotic signaling pathways, such as p38/ JNK/ p53 MAPK signaling and NF-κB signaling, which trigger the activation of pro-apoptotic factors (Redza-Dutordoir and Averill-Bates, 2016; Das et al., 2018). Oxidative stress also endorses cell death by activating cardiolipin oxidation and increase mitochondrial permeability, which trigger caspase signaling and promote myocardial cell apoptosis (Circu and Aw, 2010; Redza-Dutordoir and Averill-Bates, 2016). In our study, OEA treatment significantly attenuated Dox-mediated oxidative stress in cardiac cells, evidenced from decreased expressions of TBARS and 8-OHdG, and increased levels of GST in OEA-treated cardiomyocytes, thus improved DIC.

OEA, an endogenous fatty acid amide, is the agonist of PPARα (IC_50_ = 0.12 μM) and TRPV1 (IC_50_ = 2 μM) (Ahern, 2003; Fu et al., 2003). The biological activities of OEA, such as control of food intake, anti-inflammation, neuro-protection and lipid metabolism, have been widely investigated (Bowen et al., 2017). To date, most of these effects have been shown to be mediated by the activation of PPARα, and the role of TRPV1 in OEA-mediated actions are less-well understood (Bowen et al., 2017). In our study, the effects of OEA were likely due to activation of TRPV1 but not PPARα. Supporting this conclusion, we found that the protective effects of OEA were abolished by the TRPV1 antagonist capsazepine, but not PPARα antagonist GW6471. In addition, activation of TRPV1 but not PPARα, reduced DIC. In accordance with our observation, Mahdieh Rahmatollahi et al. have shown that antagonism of PPARα improved DOX-induced atrial dysfunction (Rahmatollahi et al., 2016). Wei Ge et al. have reported that DOX-induced cardiac functional defect and apoptosis were reversed by the TRPV1 agonist SA13353 (Ge et al., 2016).

Recent literatures have provided important insight into the connection between TRPV1 to the cell proliferation and survival (Zhai et al., 2020). Of particular important mechanism is the activation of PI3K/ Akt by TRPV1 (Zhai et al., 2020). Consistent with these observations, we found that OEA increased expressions of PI3K and p-Akt in cardiomyocytes through a TRPV1-dependent way. Moreover, previous studies have shown that activation of PI3K/ Akt ameliorates doxorubicin-induced myocardial injury (Sun et al., 2014; Liu et al., 2016; Sahu et al., 2019; Nie et al., 2021). Our data confirmed the contribution of PI3K/ Akt signaling in OEA-mediated protective effects, thereby supporting the proposed mechanism that OEA alleviates DIC through a TRPV1-mediated PI3K/ Akt pathway. The present study has several limitations. First, the cardiac function parameters were not tested in the present study. Second, the other potential limitation of the present study is that our animal model is tumor-free. As a result, we cannot rule out potential effects of OEA on the anticancer effects of DOX. Thus, future studies should be carried out to confirm this action.

In summary, our results revealed that OEA protects cardiomyocytes against DOX-induced cytotoxicity through activation of TRPV1/ PI3K/ Akt signaling. Our results also identify OEA as a promising therapy for DIC.

## Data Availability

The original contributions presented in the study are included in the article/Supplementary Material, further inquiries can be directed to the corresponding author.
