# Supplementary material for: Oleoylethanolamide as a New Therapeutic Strategy to Alleviate Doxorubicin-Induced Cardiotoxicity
Source: Front Pharmacol. 2022 Apr 20;13:863322. doi: 10.3389/fphar.2022.863322 (PMC9065409; doi:10.3389/fphar.2022.863322)
Supplement: Supplementary file 1 [file Table1.DOCX]

Oleoylethanolamide (OEA) as a new therapeutic strategy to alleviate doxorubicin (DOX)-induced cardiotoxicity (DIC)


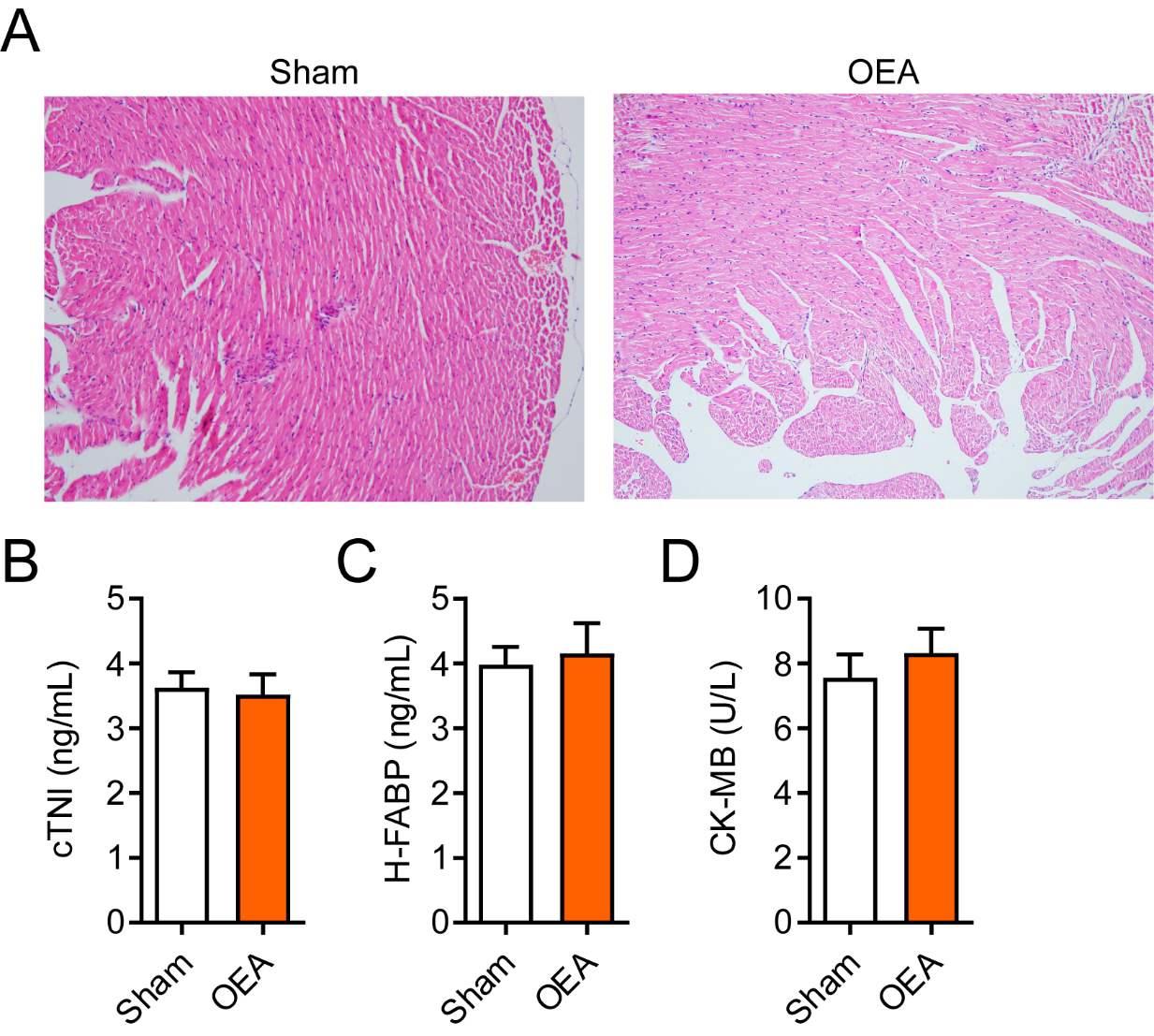


**Figure S1. Treatment with OEA alone has no effects on heart.**

Mice with or without OEA (30 mg/kg, i.v.) for 14 days. Heart tissues were isolated at day 15 for analysis. Representative histopathological sections of heart tissues by (A) Masson’s trichrome staining. The effects of OEA or its vehicle on serum levels of (B) cTNI, (C) H-FABP and (D) CK-MB in mice. Data are expressed as mean ± SEM, n = 4.


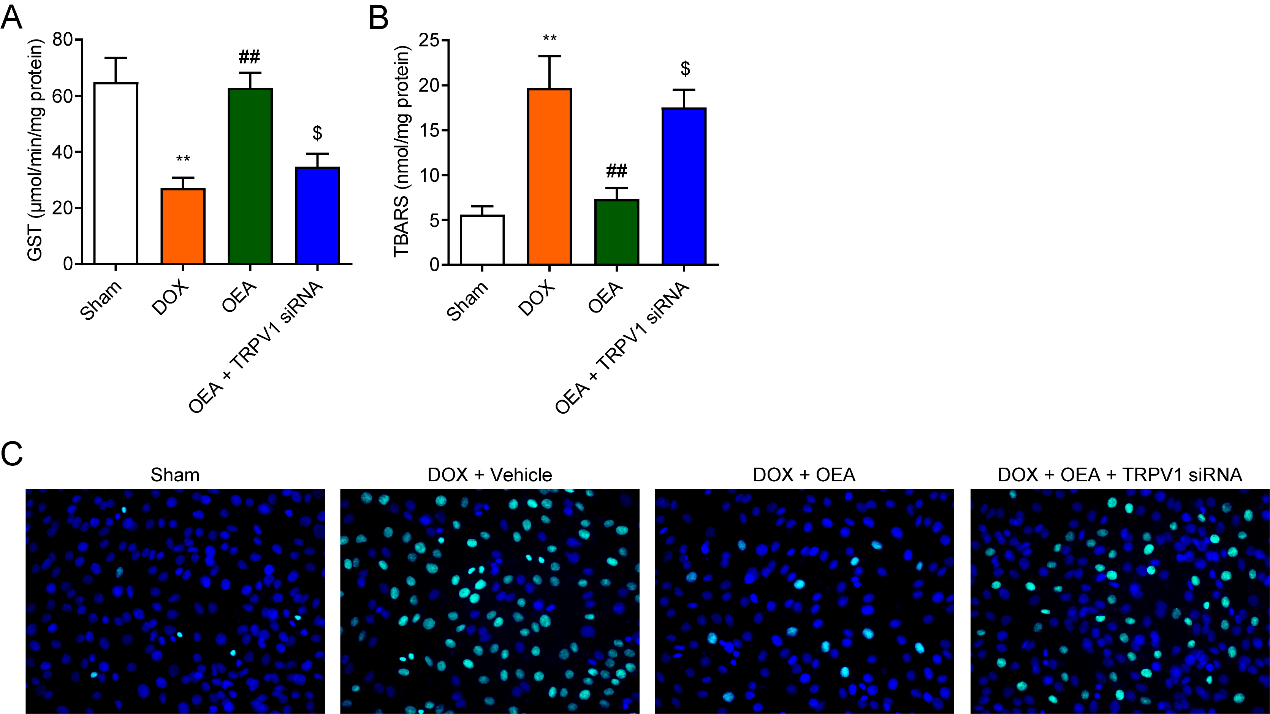


**Figure S2.** **OEA inhibited DOX-induced oxidative stress and apoptosis in** **cardiomyocytes through a TRPV1 pathway.**

HL-1 cells were treated with 0.1% DMSO and OEA (30 μM) for 30 min, or TRPV1 siRNA (30 nM) and HiPerfect transfection reagent for 18 h before challenged by DOX (2 μM) for 24 h. Levels of (A) GST and (B) TBARS was assessed in HL-1 cardiomyocytes. (C) TUNEL staining of HL-1 cardiomyocytes. Data are expressed as mean ± SEM, n = 4. **, P < 0.01 vs control. ##, P < 0.01 vs DOX + vehicle. $, P < 0.05 vs DOX + OEA.


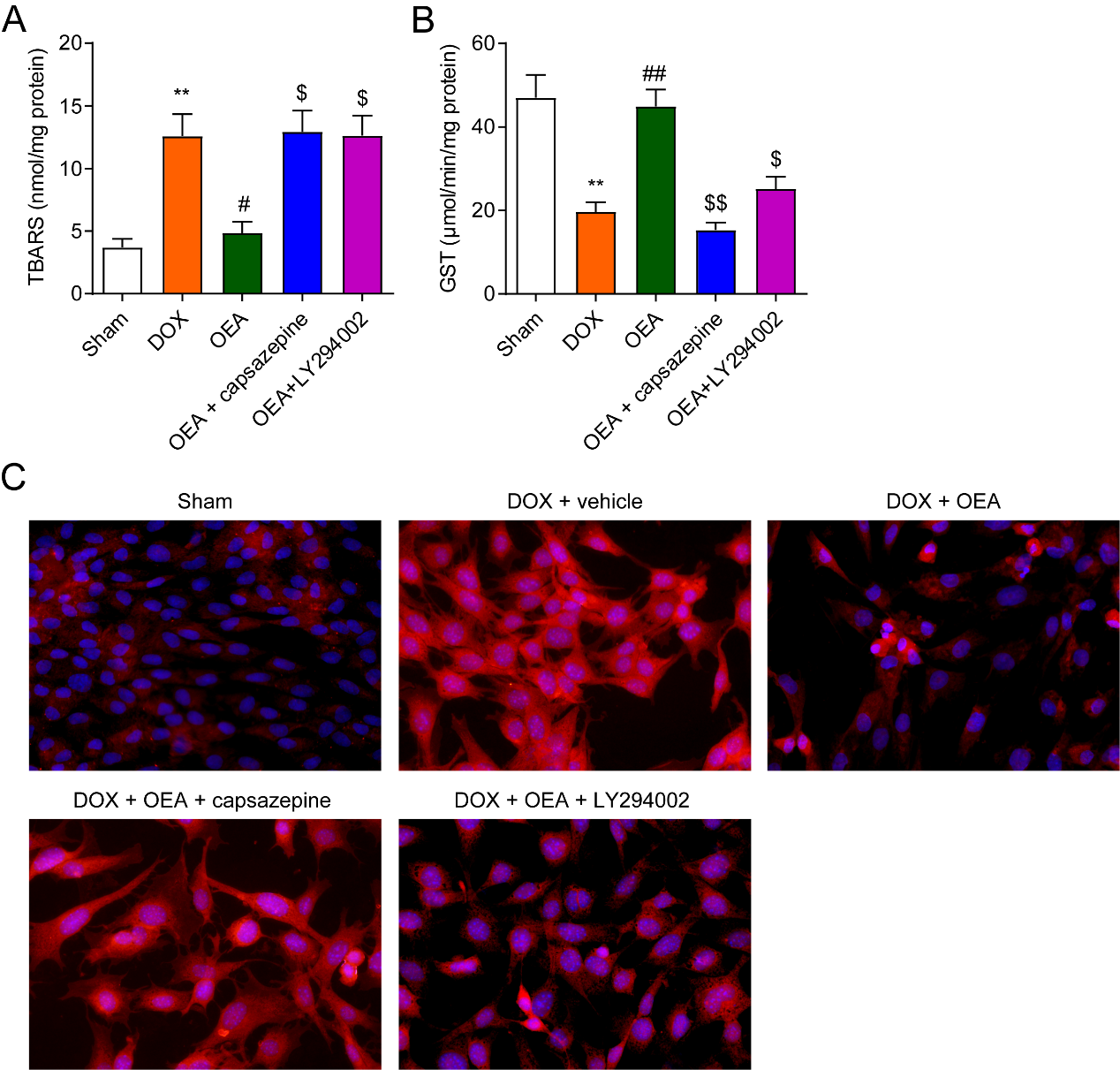


**Figure S3. Inhibition of the PI3K-Akt signaling blocks the protective effects of OEA in cardiomyocytes.**

Adult derived primary human cardiomyocytes were treated with 0.1% DMSO, OEA (30 μM), capsazepine (10 μM) and LY294002 (10 μM) for 30 min. Cells were then treated with DOX (2 μM) for 24 h. Levels of (A) GST and (B) TBARS were assessed in primary cardiomyocytes. (C) Immunofluorescence staining of caspase 3 in cardiomyocytes. Data are expressed as mean ± SEM, n = 3. ***, P < 0.001 vs control. #, P < 0.05; ##, P < 0.01; ###, P < 0.001 vs DOX + vehicle. $, P < 0.05; $$, P < 0.01; $$$, P < 0.001 vs DOX + OEA.


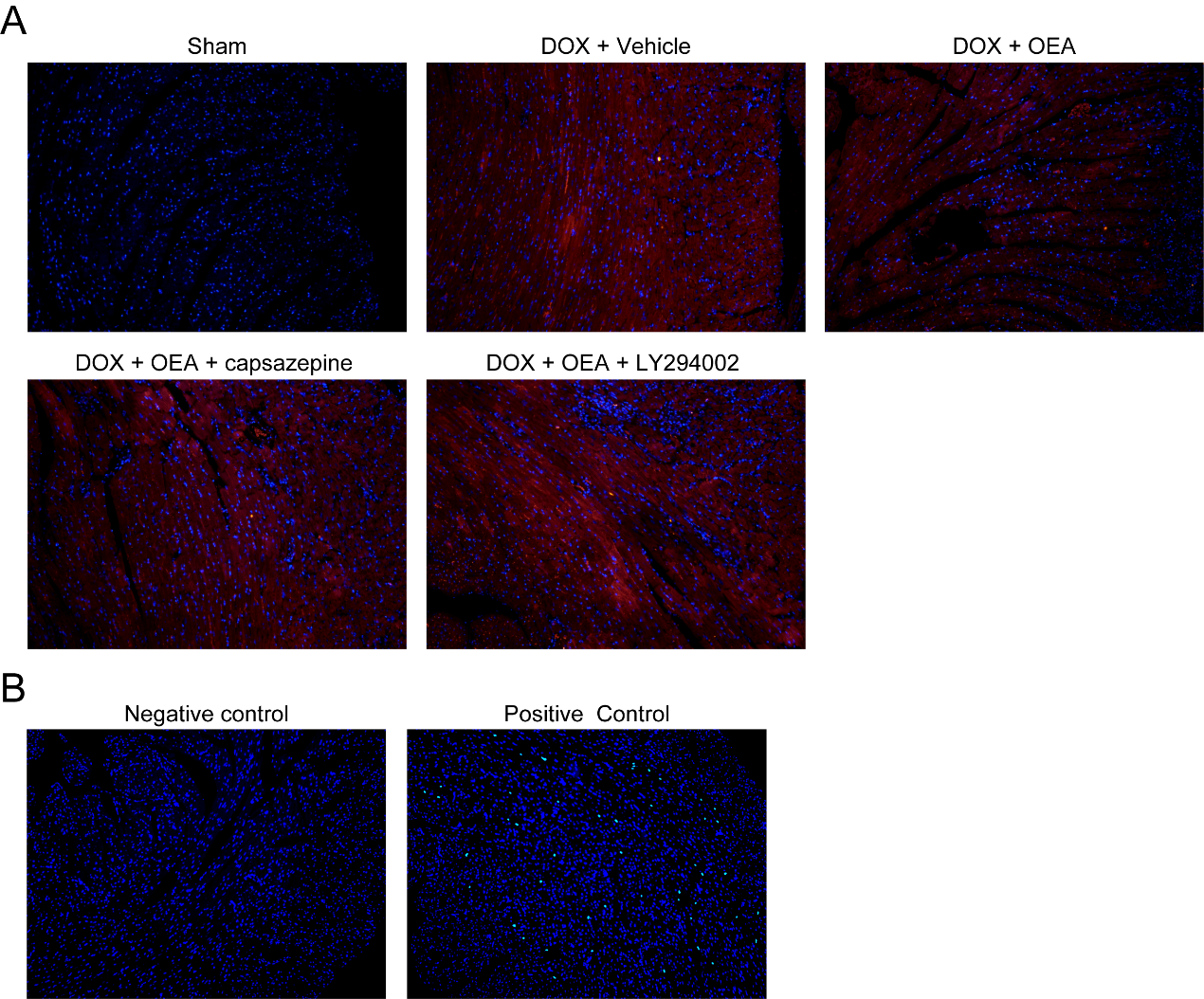


**Figure S4.** **OEA attenuates DOX-mediated myocardial oxidative stress in mice through a PI3K-dependent pathway.**

(A) Immunofluorescence staining of activated caspase 3 in myocardial tissues. Expression of caspase 3 were calculated. (B) Positive and negative controls (with secondary antibody only) for TUNEL staining.
